# Stable Clinical Course of Chronic Obstructive Pulmonary Disease Patients in the Era of Double Bronchodilator Therapy: A Single Referral Center Experience

**DOI:** 10.3390/jcm9082547

**Published:** 2020-08-06

**Authors:** Sun Hye Shin, Noeul Kang, Juhee Cho, Yeonseok Choi, Hyun Kyu Cho, Hye Sook Choi, Hojoong Kim, Jun Hyeok Lim, Hye Yun Park

**Affiliations:** 1 Division of Pulmonary and Critical Care Medicine, Department of Medicine, Samsung Medical Center, Sungkyunkwan University School of Medicine, Seoul 06351, Korea; freshsunhye@gmail.com (S.H.S.); varsagod0314@gmail.com (N.K.); yeonseokc@gmail.com (Y.C.); nara2002go@gmail.com (H.K.C.); hjk3425@skku.edu (H.K.); 2 Center for Clinical Epidemiology, Samsung Medical Center, Seoul 06351, Korea; jcho@skku.edu; 3 Department of Clinical Research Design and Evaluation, SAIHST, Sungkyunkwan University, Seoul 06355, Korea; 4 Department of Epidemiology and Welch Center for Prevention, Epidemiology, and Clinical Research, Johns Hopkins University Bloomberg School of Public Health, Baltimore, MA 21287, USA; 5 Division of Pulmonary, Allergy and Critical Care Medicine, Department of Internal Medicine, Kyung Hee University Hospital, Seoul 02447, Korea; maxymus72@hanmail.net; 6 Division of Pulmonology, Department of Internal Medicine, Inha University Hospital, Inha University School of Medicine, Incheon 22332, Korea; tboy1012@naver.com

**Keywords:** COPD, bronchodilator, inhaled corticosteroid

## Abstract

Despite clinical benefits of long-acting muscarinic antagonist (LAMA)/long-acting beta2-agonist (LABA) double bronchodilator therapy, there has been limited evidence for treatment change from LAMA/LABA to inhaled corticosteroid (ICS)-containing therapy. This study aimed to assess the rate of ICS-containing therapy from LAMA/LABA and investigate the factors associated with ICS addition. Between October 2015 and March 2019, consecutive patients prescribed with a LAMA/LABA fixed-dose combinations (FDCs) therapy without ICS were retrospectively identified from a single-referral hospital. The primary outcome was addition of ICS. During LAMA/LABA FDCs therapy (median, 12.4 months), 47 (17.7%) out of 266 patients had ICS addition. Most patients maintained bronchodilators without addition of ICS at 12 (86.5%) or 24 (76.8%) months. Patients with dyspnea (mMRC ≥ 2) at baseline, previous ICS use, and exacerbation in the previous year were at a higher risk of ICS addition. Especially, exacerbation in the previous year and dyspnea were associated with the development of frequent exacerbations during LAMA/LABA FDCs therapy, which might have led to ICS addition. Double bronchodilator therapy could be well-maintained in stable COPD patients. However, patients with exacerbation in the previous year, dyspnea, and previous ICS use should be closely approached and monitored with initiation of LAMA/LABA FDCs therapy without ICS.

## 1. Introduction

Chronic obstructive pulmonary disease (COPD) is major global health burden contributing to 3.2 million deaths annually [1]. It is characterized by persistent airflow limitation that interferes with normal breathing and causes respiratory symptoms [2]. Long-acting bronchodilators are the cornerstone in the pharmacologic treatment of symptomatic COPD patients, which are recommended as the first-line maintenance therapy to reduce symptoms and improve exercise tolerance and quality of life. Meanwhile, inhaled corticosteroids (ICSs) are reserved for patients with frequent exacerbations or one or more hospitalization due to COPD exacerbation [2].

Contrary to treatment recommendations, real-world studies demonstrated the overuse of ICS even in COPD patients with mild to moderate disease [3,4,5]. Despite a trend of a reduction in initial ICS prescriptions over time, ICS-containing therapy still accounted for approximately half the initial prescriptions in 2015 in the United Kingdom [6]. A large United States claims database study also revealed that most (75%) COPD patients receiving triple therapy had mild or moderate disease [5] and almost half of the COPD patients using ICS did not exhibit features indicative of ICS usage in 2017 from the nationwide multicenter cohorts in South Korea [7]. Furthermore, among patients prescribed with triple therapy, 25% progressed to receive triple therapy within 1 year after the diagnosis and more than 50% did so within 3 years, irrespective of the COPD severity [4].

Recently, the combination of long-acting muscarinic antagonist (LAMA) and long-acting beta2-agonist (LABA) fixed-dose combinations (FDCs) therapy was introduced and the combined use of two bronchodilators with different mechanisms is considered an effective strategy to optimize bronchodilation in COPD with a tolerable safety profile [8]. Most studies showed that LAMA/LABA FDCs therapy is more effective than LAMA or LABA monotherapy or ICS/LABA in terms of an improvement in lung function, although there were variable magnitudes of benefit regarding the improvement of dyspnea, quality of life, and prevention of exacerbation across studies [9,10,11,12,13,14,15,16,17]. In addition, the risk of pneumonia is lower in LAMA/LABA FDCs than ICS-containing inhaler regimens [17,18]. Despite these clinical benefits, data investigating the treatment evolution from LAMA/LABA FDCs therapy to ICS-containing therapy are scarce [19,20]. Therefore, the aim of this study was to assess the rate of ICS-containing therapy from LAMA/LABA FDCs and to investigate the factors associated with ICS addition in this new era of double bronchodilator therapy [21].

## 2. Materials and Methods

### 2.1. Study Population

This was a retrospective cohort study of COPD patients identified from the COPD Lung Evolution (CLUE) Registry between October 2015 and March 2019. The CLUE registry is a database of COPD patients’ records at the Samsung Medical Center (a 1979-bed referral hospital with 11 pulmonary specialists in charge of the outpatient clinic in Seoul, South Korea) that contains routinely collected anonymized data of patient demographics, comprehensive clinical parameters, laboratory and imaging studies, and prescriptions. COPD is defined as post-bronchodilator forced expiratory volume in 1s (FEV_1_)/forced vital capacity (FVC) ratio <  0.7, and patients with current asthma are excluded from the CLUE registry.

For the present study, 782 COPD patients aged 40 years or older were identified from the CLUE cohort and 376 patients who initiated LAMA/LABA FDCs during the study period were included. Patients who had been prescribed ICS at the time of LAMA/LABA FDCs initiation (N = 45), those without post-bronchodilator spirometry measurement at the time of LAMA/LABA FDCs initiation (N = 28), and those who were referred back to another hospital (N = 10) were excluded. In addition, patients with a self-reported asthma history (N = 27) were excluded. The final sample included 266 patients. The Institutional Review Board of Samsung Medical Center approved the study protocol (No. 2019-09-071-001) and waived the requirement for obtaining informed consent.

### 2.2. Double Bronchodilators

Double bronchodilator therapy with LAMA/LABA FDCs included aclidinium/formoterol (340mcg/12mcg), glycopyrronium/indacaterol (110mcg/50mcg), umeclidicium/vilanterol (62.5mcg/25mcg), and tiotropium/olodaterol (2.5mcg/2.5mcg), which are available in South Korea.

### 2.3. Data Collection and Measurements

Data obtained from the CLUE cohort database included age, sex, smoking history, body mass index (BMI), modified medical research council (mMRC) dyspnea scale, COPD assessment test (CAT) score, self-reported history of asthma, medication history including previous ICS prescription or patient-reported ICS use in the past, history of acute exacerbation, and comorbidities. Moderate exacerbation was defined as an outpatient clinic visit, while severe exacerbation was defined as hospitalization or an emergency room visit owing to one or more of the following: Worsening of dyspnea, increased sputum volume, and purulent sputum. Laboratory results included blood eosinophil counts and total immunoglobulin E (IgE). In some patients under suspicion of type 2 inflammation signatures, fractional exhaled nitric oxide (FeNO) was further conducted according to the American Thoracic Society (ATS) [22], using an NO analyzer (NIOX MINO, Aerocrine AB, Solna, Sweden) or NObreath (Bedfont Scientific, Maidstone, UK).

Spirometry and the diffusing capacity for carbon monoxide (DLco) measurements were performed using Vmax 22 (SensorMedics, Yorba Linda, CA, USA) according to the ATS/European Respiratory Society criteria [23,24]. Absolute values of FVC, FEV_1_, and DLco were obtained, and the percentage predicted values (% pred) of FVC, FEV_1_, and DLco were calculated using data obtained from a representative Korean sample [25,26]. A positive bronchodilator response was defined as a post-bronchodilator increase in FEV_1_ of at least 12% and 200 mL from the baseline value [27].

### 2.4. Outcome

The primary endpoint was the addition of ICS, as escalation to triple therapy (ICS/LAMA/LABA) or a switch to ICS/LABA from LAMA/LABA FDCs therapy. Triple therapy in a single inhaler is not yet commercially available in Korea, thus patients received triple therapy in two separate inhalers. The index date was defined as the first date on which the patient received LAMA/LABA FDCs, and the date of ICS addition or the date of the last visit without ICS addition were collected. The development of two or more exacerbations was also identified. 

### 2.5. Statistical Analysis

Data are expressed as the number (%) for categorical variables and mean (standard deviation) for continuous variables. Categorical variables were compared using the Pearson chi-square test or Fisher’s exact test, and continuous variables were compared using Student’s t-test. To estimate the rate of ICS addition after initiation of double bronchodilator therapy, the Kaplan–Meier method was used. Hazard ratios (HRs) and 95% confidence intervals (CI) for the risk of ICS addition were calculated using the Cox proportional-hazard model. Multivariable analysis for ICS addition included clinically important variables or statistically significant variables in the univariate analysis: Age, sex, smoking history (never vs. ever), BMI (< 21 vs. ≥ 21 kg/m^2^), dyspnea (mMRC ≥ 2 vs. < 2), previous ICS use, exacerbation in the previous year, COPD severity (global initiative for chronic obstructive lung disease (GOLD) grade 1,2 vs. 3,4), positive bronchodilator response, and eosinophil count ≥ 300 cells/μL. FeNO was not included in the model since it was measured in only 59 patients. In addition, multivariable logistic regression was used to investigate the factors associated with the development of frequent exacerbations (≥ 2 moderate or severe exacerbations) during double bronchodilator therapy.

All tests were two-sided, and *p*-values < 0.05 were considered significant. All analyses were performed using Stata software (ver. 14.0; Stata Corporation, College Station, TX, USA).

## 3. Results

### 3.1. Baseline Characteristics and Laboratory Findings of the Study Population

Table 1 shows the baseline characteristics of the study population. The mean age was 70.8 years, and most patients were men (92.1%) and smokers (91.0%). Never-smokers constituted 9% of the total study population. Among them, 12 patients had a previous history of pulmonary tuberculosis or bronchiectasis and 7 patients had a history of biomass exposure. Of the total, 110 (41.4%) patients had an mMRC dyspnea grade ≥ 2 and 193 (72.6%) had a CAT score ≥ 10. For 56 patients who had previously used ICS, the mean ICS duration was 825 days and the mean days from ICS discontinuation to initiation of LAMA/LABA FDCs therapy was 233 days. Most patients (43/56, 76.8%) discontinued ICS (with or without ICS dose tapering) before double bronchodilator therapy, while the remaining 13 patients (23.2%) used ICS remotely and were treated with monotherapy with either LAMA (N = 9) or LABA (N = 4) before initiating double bronchodilator therapy.

Patients who had added ICS (N = 47) were more likely to have a dyspnea of mMRC ≥ 2 (61.7% vs. 37.0%, *p* = 0.002) at the start of double bronchodilator therapy and previously used ICS (36.2% vs. 17.8%, *p* = 0.005), compared to those without ICS addition. Patient who experienced an exacerbation in the year prior to LAMA/LABA FDCs initiation were also more likely to have ICS addition (57.5% vs. 24.2%, *p* < 0.001). As shown in Table 2, post-bronchodilator FEV_1_ was significantly lower in the patients who had added ICS than in those who did not. A positive bronchodilator response, the blood eosinophil count, and total IgE did not show significant differences between the groups, while FeNO was higher in patients who added ICS (41.5 ppb vs. 25.9 ppb, *p* = 0.005).

### 3.2. Treatment Change to ICS Addition from LAMA/LABA FDCs Therapy

**Figure 1:**
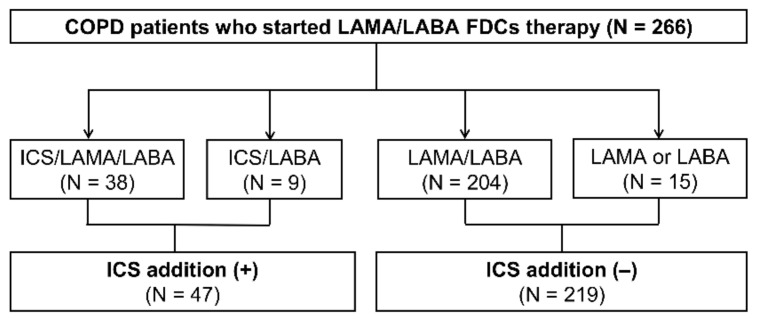
Treatment evolution after initiation of LAMA/LABA fixed-dose combinations therapy. CAT, COPD assessment test; FDC, fixed-dose combinations; ICS, inhaled corticosteroids; LABA, long-acting beta2-agonist; LAMA, long-acting muscarinic antagonist.

In the median follow-up period of 12.4 (interquartile range, 5.6–22.2) months, 47 (17.7%) patients added ICS, 204 (76.7%) maintained LAMA/LABA FDCs therapy, and 15 (5.6%) de-escalated to monotherapy. Among the 47 patients who added ICS, 38 (14.3%) escalated to triple therapy and 9 (3.4%) changed to ICS/LABA (Figure 1).

**Figure 2:**
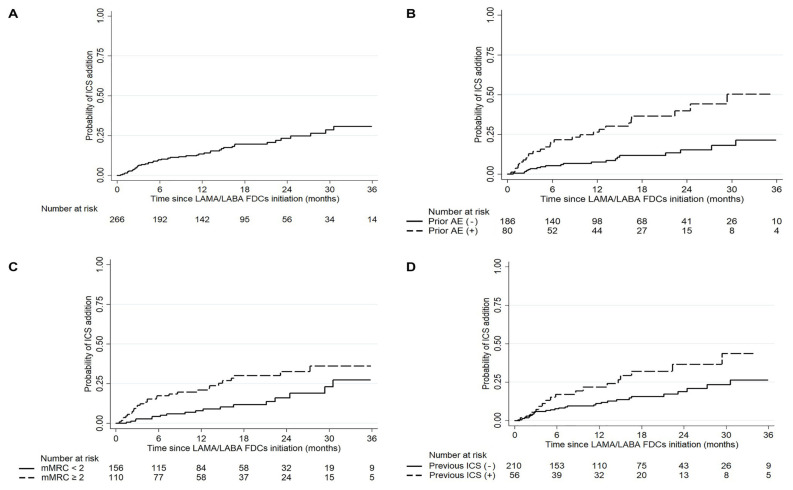
Kaplan–Meier curves for ICS addition in (**A**) overall patients and by (**B**) exacerbation in the previous year, (**C**) baseline dyspnea of mMRC ≥ 2, and (**D**) previous ICS use. FDC, fixed-dose combinations; ICS, inhaled corticosteroids; Prior AE, acute exacerbation in the previous year; LABA, long-acting beta2-agonist; LAMA, long-acting muscarinic antagonist; mMRC, modified medical research council.

As shown in Figure 2A, only 13.5% and 23.2% patients had added ICS at 12 and 24 months after initiating double bronchodilator therapy. Among those who maintained LAMA/LABA FDCs therapy for more than 90 days, 8.0% and 18.4% added ICS at 12 and 24 months, respectively (Supplementary Figure S1). When patients were divided according to study periods (those who started double bronchodilators before and after January 1, 2017, respectively), we found the trend of ICS addition was not different between the two groups (log-rank *p* = 0.071, Supplementary Figure S2).

### 3.3. Clinical Factors Associated with ICS Addition after Initiation of LAMA/LABA FDCs Therapy

In multivariable models, dyspnea at baseline (mMRC ≥ 2) (adjusted HR [aHR], 3.38; 95% CI, 1.73–6.63), previous ICS use (aHR, 2.04; 95% CI, 1.00–4.15), and exacerbation in the previous year (aHR, 3.97; 95% CI, 2.13–7.40) were independently associated with the risk of ICS addition after initiation of LAMA/LABA FDCs therapy (Table 3).

In the Kaplan–Meier analysis of ICS addition according to previous exacerbation, dyspnea at baseline, and previous ICS use (Figure 2B–D, respectively), most patients without these risk factors did not add ICS at 12 or 24 months, which showed a marked difference from those who had any of these factors (log-rank *p* < 0.05 in all comparisons).

In the sensitivity analysis in 108 treatment-naïve patients (Supplementary Table S1), only the exacerbation history in the previous year retained a significant association with ICS addition (aHR, 11.57; 95% CI 3.24 – 41.38). When ICS withdrawal before double bronchodilator therapy (N = 43) was included in the multivariable analysis instead of the entire previous ICS use (Supplementary Table S2), a stronger association between ICS withdrawal and the ICS addition after initiation of LAMA/LABA FDCs therapy (aHR, 2.70; 95% CI 1.32–5.53) was observed.

### 3.4. Clinical Factors Associated with Frequent Exacerbations During LAMA/LABA FDCs Therapy

During LAMA/LABA FDCs therapy, 26 (9.8%) patients experienced ≥ 2 moderate or severe exacerbations, which were more prevalent in patients with ICS addition than in those without ICS addition (31.9% vs. 5.0%, *p* < 0.001). Among 26 patients, 5 had severe exacerbations and 21 had moderate exacerbations. As shown in Table 4, the dyspnea at baseline (mMRC ≥ 2) (adjusted odds ratio [aOR], 2.64; 95% CI, 1.03–6.74) and history of exacerbation in the previous year (aOR, 5.28; 95% CI, 2.16–12.92) were associated with the development of ≥ 2 exacerbations during LAMA/LABA FDCs therapy.

## 4. Discussion

In the present study, among 266 COPD patients with double bronchodilator therapy of LAMA/LABA FDCs, we found that 87% of patients maintained bronchodilator therapy without adding ICS at 12 months and 77% of patients persisted with bronchodilator therapy without adding ICS at 24 months. Particularly, among those who had not used ICS prior to LAMA/LABA FDCs initiation, 81% remained without ICS addition until 24 months. Factors associated with ICS addition were exacerbation in the previous year, dyspnea of mMRC ≥ 2 at baseline, and previous ICS use before LAMA/LABA FDCs therapy. Especially, exacerbation in the previous year and dyspnea of mMRC ≥ 2 at baseline were associated with the development of frequent exacerbations during LAMA/LABA FDCs treatment, which might lead to ICS addition following LAMA/LABA FDCs.

In our study, the proportions of ICS addition (escalation to triple therapy in most cases and change to ICS/LABA in some cases) within 12 and 24 months of commencing LAMA/LABA FDCs treatment were 14% and 23%, respectively, which were lower than the results from previous studies that investigated the escalation to triple therapy from ICS/LABA or LAMA. In a previous study based on a United Kingdom primary setting, 26.8% of the patients who started with LAMA monotherapy progressed to triple therapy within 12 months [28]. Likewise, in a study using the United States health care claims data, more than 50% of patients who used LAMA monotherapy (median 244 days) or the ICS/LABA combination (median 281 days) progressed to triple therapy within 12 months [20]. In a recent study published after LAMA/LABA FDCs became available, patients receiving LAMA/LABA FDC (umeclidinium/vilanterol) were at a lower risk of progression to triple therapy than those receiving LAMA (tiotropium), with a longer mean time to triple therapy (6.5 vs. 5.6 months) [19], but this study lacked data for symptoms and lung function because of the nature of claims data. Taken together, escalation to triple therapy could be delayed in COPD patients receiving the LAMA/LABA FDCs in this new era of double bronchodilator therapy.

We further investigated the baseline factors associated with ICS addition after LAMA/LABA FDCs therapy and found that exacerbation in the previous year strongly influenced ICS addition. This result is in line with a previous study [4] and a history of exacerbations is a well-known factor predicting future exacerbations [29]. Furthermore, in our study, patients with more severe dyspnea, indicated by an mMRC grade ≥ 2 at baseline, were prone to adding ICS during LAMA/LABA FDCs therapy. In a post hoc analysis of the large-numbered clinical trial, a higher mMRC grade at baseline was associated with the future exacerbation risk in a dose-dependent manner, especially when mMRC ≥ 2 [30]. Another analysis using four RCTs also showed a significantly decreased risk of exacerbations with triple therapy as compared with double therapy with LABA/LAMA in symptomatic patients [31], which was persistent even in the subgroup analysis, with less than one exacerbation in the previous year [32]. Considering that dyspnea perception is enhanced in patients with frequent exacerbation and blunted in those with infrequent exacerbations [33], addition of ICS having an anti-inflammatory effect in COPD patients could influence the perception of dyspnea with a reduction of future exacerbations [34,35]. Indeed, our study showed that these clinical factors (previous exacerbation and dyspnea) were also associated with the development of frequent exacerbation during double bronchodilator therapy. Frequent exacerbations, in turn, might have strongly influenced the treatment decision of ICS addition. Thus, when double bronchodilators are selected as an initial therapy in COPD patients, the possibility of ICS addition afterwards should be considered in those with exacerbations in the previous year or dyspnea at baseline.

Patients who previously used ICS were more likely to have ICS addition during LAMA/LABA FDCs therapy as well. Indeed, 22% of the patients with previous ICS use eventually received ICS again within 12 months of LAMA/LABA FDCs commencement. However, previous ICS itself was not associated with the development of frequent exacerbation during double bronchodilator therapy in our study. When we further looked into the data, among 56 patients with previous ICS use, most patients (n = 43) had ICS withdrawal just before double bronchodilator therapy and the remaining 13 patients had used ICS in the past but were receiving monotherapy ahead of double bronchodilator therapy. The sensitivity analysis showed a stronger association between ICS withdrawal and the ICS addition than the analysis using any ICS use in the past (Supplementary Table S2), suggesting that the decision of ICS addition during double bronchodilator therapy might have been affected by the physician’s tendency to restart ICS. Moreover, 27 out of 43 patients (62.8%) maintained LAMA/LABA FDCs therapy (n = 26) or even de-escalated to monotherapy (n = 1). Thus, ICS withdrawal can still be considered in patients who have no apparent indications for ICS [36,37], with close monitoring required for patients who previously used ICS.

The Global Initiative for Chronic Obstructive Lung Disease 2019 report recommended using the absolute blood eosinophil count with a threshold of 300 cells/μL as a guide for ICS use in stable COPD patients having exacerbations [38]. In our study, 15% of the patients had an eosinophil count ≥ 300 cells/μL when they commenced LAMA/LABA FDCs therapy. However, there was no significant difference in the blood eosinophil count between the patients who had added ICS and those who had not, in line with the FLAME study. In FLAME study, 80% of patients had one moderate exacerbation and 20% of those had ≥ 2 moderate exacerbations in the previous year, and the LAMA/LABA FDC therapy was more effective in exacerbation prevention compared to ICS/LABA, irrespective of the blood eosinophil count [39]. However, this was inconsistent with the IMPACT study [40], which had 71% of COPD patients with ≥ 2 moderate or ≥ 1 severe exacerbations in the previous year, and showed that a high blood eosinophil count was associated with the benefit of triple therapy over double therapy. Given that 70% of our study population had no history of exacerbation in the previous year, the characteristics of the study population might affect the role of eosinophil levels in predicting the response to ICS. Regarding FeNO, another measurement of eosinophilic airway inflammation, its utility is still unclear in COPD, unlike asthma. In our study, FeNO was measured in 59 out of 266 patients and its level was higher in patients who added ICS than those who maintained LAMA/LABA FDCs therapy. However, given that FeNO was measured in only 22% of our study population, more evidence is needed to clarify the role of FeNO for ICS addition in COPD patients with LAMA/LABA FCDs therapy.

Lastly, as the GOLD 2017 report updated the overall management of COPD with only respiratory symptoms and exacerbations for COPD assessment, subgroup analysis was conducted with two groups (those who started double bronchodilators before and after January 1, 2017, respectively) and we found the trend of ICS addition did not differ by study periods (Supplementary Figure S2). As shown in Table 3, ICS addition was not associated with baseline FEV1 <50% (GOLD grade ≥ 3), but it was associated with patients’ symptoms and exacerbation history. In this regard, the trend of ICS addition may not have been influenced by the change in the GOLD 2017 report.

There are potential limitations to this study. First, this was a retrospective cohort study conducted at a single-referral hospital, which could limit the generalizability of our results. Furthermore, all study subjects were prescribed with LAMA/LABA FDCs without ICS at baseline, and those who initiated or continued with the ICS-containing regimen were not included in this study. Therefore, the findings of our study cannot be generalized as the outcome of LAMA/LABA FDCs therapy in the entire COPD population. Secondly, we could not make a comparison with monotherapy or ICS/LABA for the duration of the treatment maintenance before ICS addition. However, the duration of LAMA/LABA FDCs cannot be evaluated in randomized controlled studies because randomized medications have to be used until the end of the study unless participants dropped out from the trial. In addition, comparative studies using health care claims data lacked data regarding the reasons for ICS addition due to the nature of claims data [19,20]. Thus, our study reflecting the real-world practice of COPD would be informative to clinicians who will initiate LAMA/LABA FDCs. Finally, when we analyzed the lung function change during LAMA/LABA FDCs therapy, patients who added ICS showed a greater mean annual FEV_1_ decline (mL/year) compared to those who maintained bronchodilators, which did not reach the statistical significance (Supplementary Table S3) even after adjustment for the same variables in Table 4. However, the mean follow-up in our study was 12 months, which is relatively short to evaluate lung function change. Prospective studies with a longer follow-up duration and long-term clinical outcomes, such as lung function decline, are necessary to confirm our findings.

## 5. Conclusions

Double bronchodilator therapy with an LAMA/LABA FDCs could be well-maintained in stable COPD patients. However, patients with exacerbation in the previous year, dyspnea of mMRC ≥ 2 at baseline, and previous ICS use before LAMA/LABA FDCs therapy should be closely approached and monitored with initiation of LAMA/LABA FDCs therapy without ICS.

## Figures and Tables

**Table 1:** Baseline characteristics of 266 patients according to ICS addition after LAMA/LABA fixed-dose combinations therapy.

	Total (N = 266)	ICS Addition (-) (N = 219)	ICS Addition (+) (N = 47)	*p*-value
Age, year	70.8 (8.3)	70.7 (8.6)	71.4 (6.8)	0.579
Sex, male	245 (92.1)	202 (92.2)	43 (91.5)	0.772
Smoking status				0.672
Current	93 (35.0)	74 (33.8)	19 (40.4)	
Former	149 (56.0)	125 (57.1)	24 (51.1)	
Never	24 (9.0)	20 (9.1)	4 (8.5)	
BMI < 21 (kg/m^2^)	59 (22.2)	50 (22.8)	9 (19.2)	0.581
mMRC ≥ 2	110 (41.4)	81 (37.0)	29 (61.7)	**0.002**
CAT score ≥ 10	193 (72.6)	155 (70.8)	38 (80.9)	0.160
Previous ICS use	56 (21.1)	39 (17.8)	17 (36.2)	**0.005**
Exacerbation * in the previous year	80 (30.1)	53 (24.2)	27 (57.5)	**<0.001**
Comorbidities				
History of PTB	9 (3.4)	5 (2.3)	4 (8.5)	0.055
Lung cancer	8 (3.0)	7 (3.2)	1 (2.1)	1.000
Any	101 (38.0)	85 (38.8)	16 (34.0)	0.541

Values are mean (standard deviation) or number (%). * Exacerbation of moderate (hospital visit) or severe (emergency department or hospitalization) degree. BMI, body mass index; CAT, COPD assessment test; ICS, inhaled corticosteroids; LABA, long-acting beta2-agonist; LAMA, long-acting muscarinic antagonist; mMRC, modified medical research council; PTB, pulmonary tuberculosis.

**Table 2:** Pulmonary function test and laboratory findings of 266 patients according to ICS addition after LAMA/LABA fixed-dose combinations therapy.

	Total (N = 266)	ICS Addition (-) (N = 219)	ICS Addition (+) (N = 47)	*p*-Value
Post-bronchodilator spirometry				
FVC, L	3.35 (0.78)	3.37 (0.75)	3.25 (0.89)	0.314
FVC, % pred	80.4 (15.2)	80.6 (14.9)	79.2 (16.6)	0.565
FEV_1_, L	1.81 (0.48)	1.86 (0.47)	1.62 (0.51)	**0.003**
FEV_1_, %pred	62.5 (13.1)	63.6 (12.6)	57.3 (14.1)	**0.003**
GOLD grade 1	21 (7.9)	20 (9.1)	1 (2.1)	**0.033**
GOLD grade 2	202 (75.9)	168 (76.7)	34 (72.3)	
GOLD grade 3	40 (15.0)	30 (13.7)	10 (21.3)	
GOLD grade 4	3 (1.1)	1 (0.5)	2 (4.3)	
FEV_1_/FVC	54.8 (9.9)	55.5 (9.4)	51.4 (11.6)	**0.009**
Positive BDR	54 (20.3)	43 (19.6)	11 (23.4)	0.560
DLco, % pred (n = 232)	64.4 (19.7)	65.4 (19.6)	59.9 (19.7)	0.101
≥ 80%	56 (24.1)	49 (25.8)	7 (16.7)	0.211
< 80%	176 (75.9)	141 (74.2)	35 (83.3)	
Blood eosinophil count (/μL) (n = 264)	218.1 (369.1)	217.5 (398.4)	221.1 (166.7)	0.952
< 300	224 (84.9)	185 (84.5)	39 (83.0)	0.709
≥ 300	40 (15.1)	34 (15.5)	6 (13.3)	
FeNO (ppb) (n = 59)	29.3 (18.1)	25.9 (13.0)	41.5 (27.3)	**0.005**
Total IgE (kU/L) (n = 223)	235.1 (384.2)	232.4 (371.5)	247.4 (443.0)	0.824

Values are mean (standard deviation) or number (%). COPD severity was classified according to the Global Initiative for Chronic Obstructive Lung Disease (GOLD) grading system as GOLD grade 1 (FEV_1_ ≥ 80% predicted), GOLD grade 2 (50% ≤ FEV_1_ < 80% predicted), GOLD grade 3 (30% ≤ FEV_1_ < 50% predicted), or GOLD grade 4 (FEV1 < 30% predicted). BDR, bronchodilator response; DLco, diffusing capacity for carbon monoxide; FeNO, fractional exhaled nitric oxide; FEV_1_, forced expiratory volume in 1s; FVC, forced vital capacity; GOLD, global initiative for chronic obstructive lung disease; ICS, inhaled corticosteroids; IgE, immunoglobulin E; LABA, long-acting beta2-agonist; LAMA, long-acting muscarinic antagonist.

**Table 3:** Clinical factors associated with ICS addition after LAMA/LABA fixed-dose combinations therapy.

	Univariate Analysis		Multivariate Analysis	
	Unadjusted HR (95% CI)	*p*-Value	Adjusted HR (95% CI)	*p*-Value
Age, years	1.01 (0.98–1.05)	0.460	0.99 (0.95–1.03)	0.719
Male sex	0.88 (0.32–2.46)	0.813	0.48 (0.09–2.57)	0.392
Smoking, ever	1.10 (0.39–3.06)	0.857	1.64 (0.31–8.68)	0.558
BMI < 21(kg/m^2^)	0.97 (0.47–2.00)	0.927	0.69 (0.30–1.60)	0.393
mMRC ≥ 2	2.31 (1.28–4.16)	**0.005**	3.38 (1.73–6.63)	**<0.001**
CAT ≥ 10	1.69 (0.82–3.50)	0.157		
Previous ICS use	2.03 (1.12–3.68)	**0.020**	2.04 (1.00–4.15)	**0.049**
Exacerbation in the previous year *	3.39 (1.90–6.04)	**<0.001**	3.97 (2.13–7.40)	**<0.001**
GOLD grade ≥ 3 †	1.80 (0.93–3.47)	0.080	1.50 (0.73–3.08)	0.268
DLco < 80% pred	1.87 (0.83–4.21)	0.132		
Positive BDR	1.36 (0.69–2.67)	0.376	1.66 (0.82–3.38)	0.162
Blood eosinophil count ≥ 300 (/μL)	0.91 (0.39–2.15)	0.833	1.14 (0.47–2.77)	0.765

* Exacerbation of moderate (hospital visit) or severe (emergency department or hospitalization) degree. † GOLD ≥ grade 3 (FEV_1_ < 50% predicted). BDR, bronchodilator response; BMI, body mass index; CAT, COPD assessment test; CI, confidence interval; DLco, diffusing capacity for carbon monoxide; FEV_1_, forced expiratory volume in 1s; GOLD, global initiative for chronic obstructive lung disease; HR, hazard ratios; ICS, inhaled corticosteroids; LABA, long-acting beta2-agonist; LAMA, long-acting muscarinic antagonist; mMRC, modified medical research council.

**Table 4:** Clinical factors associated with ≥ 2 exacerbations * during LAMA/LABA fixed-dose combinations therapy.

	Univariate Analysis		Multivariate Analysis	
	Unadjusted OR (95% CI)	*p*-value	Adjusted OR (95% CI)	*p*-value
Age, years	1.02 (0.97–1.07)	0.543	1.00 (0.94–1.05)	0.867
Male sex †	1.00 (N/A)	N/A		
Smoking, ever	2.65 (0.34–20.47)	0.350	3.60 (0.44–29.30)	0.232
BMI < 21(kg/m^2^)	0.27 (0.06–1.17)	0.079	0.22 (0.05–1.01)	0.052
mMRC ≥ 2	2.08 (0.92–4.73)	0.080	2.64 (1.03–6.74)	**0.043**
CAT ≥ 10	1.66 (0.60–4.58)	0.327		
Previous ICS use	2.17 (0.91–5.18)	0.080	2.00 (0.74–5.41)	0.170
Exacerbation in the previous year	5.31 (2.25–12.51)	**<0.001**	5.28 (2.16–12.92)	**<0.001**
GOLD grade ≥ 3 ‡	1.65 (0.62–4.37)	0.318	1.38 (0.47–4.07)	0.563
DLco < 80% pred	1.57 (0.51–4.84)	0.429		

* Exacerbation of moderate (hospital visit) or severe (emergency department or hospitalization) degree. † All 26 patients who had ≥ 2 exacerbations were male. ‡ GOLD ≥ grade 3 (FEV_1_ < 50% predicted). BDR, bronchodilator response; BMI, body mass index; CAT, COPD assessment test; CI, confidence interval; DLco, diffusing capacity for carbon monoxide; FEV_1_, forced expiratory volume in 1s; GOLD, global initiative for chronic obstructive lung disease; HR, hazard ratios; ICS, inhaled corticosteroids; LABA, long-acting beta2-agonist; LAMA, long-acting muscarinic antagonist; mMRC, modified medical research council.

## Supplementary Materials

The following are available online at https://www.mdpi.com/2077-0383/9/8/2547/s1, Figure S1: Kaplan-Meier curves for ICS addition in 234 patients who maintained LAMA/LABA fixed-dose combinations therapy for more than 90 days; Figure S2: Kaplan–Meier curves for ICS addition in patients who started LAMA/LABA fixed-dose combinations therapy before and after January 1, 2017, Table S1: Clinical factors associated with ICS addition after LAMA/LABA fixed-dose combinations therapy including only treatment naïve patients; Supplementary Table S2. Clinical factors associated with ICS addition after LAMA/LABA fixed-dose combinations therapy; Table S3. Changes in pulmonary function from baseline to the end of LAMA/LABA fixed-dose combinations therapy according to ICS addition afterwards.

## References

[1] Collaborators G.B.D.C.R.D. (2017). Global, regional, and national deaths, prevalence, disability-adjusted life years, and years lived with disability for chronic obstructive pulmonary disease and asthma, 1990–2015: A systematic analysis for the Global Burden of Disease Study 2015. Lancet Respir. Med..

[2] Global Initiative for Chronic Obstructive Lung Disease Global Strategy for the Diagnosis, Management, and Prevention of Chronic Obstructive Pulmonary Disease: 2020 REPORT. http://www.goldcopd.org.

[3] Price D., West D., Brusselle G., Gruffydd-Jones K., Jones R., Miravitlles M., Rossi A., Hutton C., Ashton V.L., Stewart R. (2014). Management of COPD in the UK primary-care setting: An analysis of real-life prescribing patterns. Int. J. Chronic Obstr. Pulm. Dis..

[4] Brusselle G., Price D., Gruffydd-Jones K., Miravitlles M., Keininger D.L., Stewart R., Baldwin M., Jones R.C. (2015). The inevitable drift to triple therapy in COPD: An analysis of prescribing pathways in the UK. Int. J. Chronic Obstr. Pulm. Dis..

[5] Simeone J.C., Luthra R., Kaila S., Pan X., Bhagnani T.D., Liu J., Wilcox T.K. (2017). Initiation of triple therapy maintenance treatment among patients with COPD in the US. Int. J. Chronic Obstr. Pulm. Dis..

[6] Chalmers J.D., Tebboth A., Gayle A., Ternouth A., Ramscar N. (2017). Determinants of initial inhaled corticosteroid use in patients with GOLD A/B COPD: A retrospective study of UK general practice. NPJ Prim. Care Respir. Med..

[7] Lee S.H., Lee J.H., Yoon H.I., Park H.Y., Kim T.H., Yoo K.H., Oh Y.M., Jung K.S., Lee S.D., Lee S.W. (2019). Change in inhaled corticosteroid treatment and COPD exacerbations: An analysis of real-world data from the KOLD/KOCOSS cohorts. Respir. Res..

[8] Calzetta L., Rogliani P., Matera M.G., Cazzola M. (2016). A Systematic Review With Meta-Analysis of Dual Bronchodilation With LAMA/LABA for the Treatment of Stable COPD. Chest.

[9] Bateman E.D., Ferguson G.T., Barnes N., Gallagher N., Green Y., Henley M., Banerji D. (2013). Dual bronchodilation with QVA149 versus single bronchodilator therapy: The SHINE study. Eur. Respir. J..

[10] Wedzicha J.A., Decramer M., Ficker J.H., Niewoehner D.E., Sandstrom T., Taylor A.F., D’Andrea P., Arrasate C., Chen H., Banerji D. (2013). Analysis of chronic obstructive pulmonary disease exacerbations with the dual bronchodilator QVA149 compared with glycopyrronium and tiotropium (SPARK): A randomised, double-blind, parallel-group study. Lancet Respir. Med..

[11] Celli B., Crater G., Kilbride S., Mehta R., Tabberer M., Kalberg C.J., Church A. (2014). Once-daily umeclidinium/vilanterol 125/25 mcg in COPD: A randomized, controlled study. Chest.

[12] Decramer M., Anzueto A., Kerwin E., Kaelin T., Richard N., Crater G., Tabberer M., Harris S., Church A. (2014). Efficacy and safety of umeclidinium plus vilanterol versus tiotropium, vilanterol, or umeclidinium monotherapies over 24 weeks in patients with chronic obstructive pulmonary disease: Results from two multicentre, blinded, randomised controlled trials. Lancet Respir. Med..

[13] Singh D., Jones P.W., Bateman E.D., Korn S., Serra C., Molins E., Caracta C., Gil E.G., Leselbaum A. (2014). Efficacy and safety of aclidinium bromide/formoterol fumarate fixed-dose combinations compared with individual components and placebo in patients with COPD (ACLIFORM-COPD): A multicentre, randomised study. BMC Pulm. Med..

[14] Buhl R., Maltais F., Abrahams R., Bjermer L., Derom E., Ferguson G., Flezar M., Hebert J., McGarvey L., Pizzichini E. (2015). Tiotropium and olodaterol fixed-dose combination versus mono-components in COPD (GOLD 2–4). Eur. Respir. J..

[15] O’Donnell D.E., Casaburi R., Frith P., Kirsten A., De Sousa D., Hamilton A., Xue W., Maltais F. (2017). Effects of combined tiotropium/olodaterol on inspiratory capacity and exercise endurance in COPD. Eur. Respir. J..

[16] Maltais F., Bjermer L., Kerwin E.M., Jones P.W., Watkins M.L., Tombs L., Naya I.P., Boucot I.H., Lipson D.A., Compton C. (2019). Efficacy of umeclidinium/vilanterol versus umeclidinium and salmeterol monotherapies in symptomatic patients with COPD not receiving inhaled corticosteroids: The EMAX randomised trial. Respir. Res..

[17] Horita N., Goto A., Shibata Y., Ota E., Nakashima K., Nagai K., Kaneko T. (2017). Long-acting muscarinic antagonist (LAMA) plus long-acting beta-agonist (LABA) versus LABA plus inhaled corticosteroid (ICS) for stable chronic obstructive pulmonary disease (COPD). Cochrane Database Syst. Rev..

[18] Suissa S., Patenaude V., Lapi F., Ernst P. (2013). Inhaled corticosteroids in COPD and the risk of serious pneumonia. Thorax.

[19] Hahn B., Hull M., Blauer-Peterson C., Buikema A.R., Ray R., Stanford R.H. (2018). Rates of escalation to triple COPD therapy among incident users of LAMA and LAMA/LABA. Respir. Med..

[20] Lane D.C., Stemkowski S., Stanford R.H., Tao Z. (2018). Initiation of Triple Therapy with Multiple Inhalers in Chronic Obstructive Pulmonary Disease: An Analysis of Treatment Patterns from a U.S. Retrospective Database Study. J. Manag. Care Spec. Pharm..

[21] López-Campos J.L., Abad-Arranz M., Calero-Acuña C. (2015). Double or Dual Bronchodilation: Defining the Correct Term. Arch. Bronconeumol..

[22] Dweik R.A., Boggs P.B., Erzurum S.C., Irvin C.G., Leigh M.W., Lundberg J.O., Olin A.C., Plummer A.L., Taylor D.R. (2011). An official ATS clinical practice guideline: Interpretation of exhaled nitric oxide levels (FENO) for clinical applications. Am. J. Respir. Crit. Care Med..

[23] Miller M. (2005). ATS/ERS task force: Standardisation of spirometry. Eur. Respir. J..

[24] American Thoracic Society (1995). Single-breath carbon monoxide diffusing capacity (transfer factor). Recommendations for a standard technique-1995 update. Am. J. Respir. Crit. Care Med..

[25] Choi J.K., Paek D., Lee J.O. (2005). Normal predictive values of spirometry in Korean population. Tuberc. Respir. Dis..

[26] Park J., Choi I., Park K. (1985). Normal predicted standards of single breath carbon monoxide diffusing capacity of lung in healthy nonsmoking adults. Korean J. Intern. Med..

[27] Pellegrino R., Viegi G., Brusasco V., Crapo R.O., Burgos F., Casaburi R., Coates A., van der Grinten C.P., Gustafsson P., Hankinson J. (2005). Interpretative strategies for lung function tests. Eur. Respir. J..

[28] Wurst K.E., Punekar Y.S., Shukla A. (2014). Treatment evolution after COPD diagnosis in the UK primary care setting. PLoS ONE.

[29] Hurst J.R., Vestbo J., Anzueto A., Locantore N., Mullerova H., Tal-Singer R., Miller B., Lomas D.A., Agusti A., Macnee W. (2010). Susceptibility to exacerbation in chronic obstructive pulmonary disease. N. Engl. J. Med..

[30] Calverley P.M., Tetzlaff K., Dusser D., Wise R.A., Mueller A., Metzdorf N., Anzueto A. (2017). Determinants of exacerbation risk in patients with COPD in the TIOSPIR study. Int. J. Chronic Obstr. Pulm. Dis..

[31] Nici L., Mammen M.J., Charbek E., Alexander P.E., Au D.H., Boyd C.M., Criner G.J., Donaldson G.C., Dreher M., Fan V.S. (2020). Pharmacologic Management of Chronic Obstructive Pulmonary Disease. An Official American Thoracic Society Clinical Practice Guideline. Am. J. Respir. Crit. Care Med..

[32] Ferguson G.T., Rabe K.F., Martinez F.J., Fabbri L.M., Wang C., Ichinose M., Bourne E., Ballal S., Darken P., DeAngelis K. (2018). Triple therapy with budesonide/glycopyrrolate/formoterol fumarate with co-suspension delivery technology versus dual therapies in chronic obstructive pulmonary disease (KRONOS): A double-blind, parallel-group, multicentre, phase 3 randomised controlled trial. Lancet Respir. Med..

[33] Scioscia G., Blanco I., Arismendi E., Burgos F., Gistau C., Foschino Barbaro M.P., Celli B., O’Donnell D.E., Agustí A. (2017). Different dyspnoea perception in COPD patients with frequent and infrequent exacerbations. Thorax.

[34] Von Leupoldt A., Kanniess F., Dahme B. (2007). The influence of corticosteroids on the perception of dyspnea in asthma. Respir. Med..

[35] Rabe K.F. (2006). Improving dyspnea in chronic obstructive pulmonary disease: Optimal treatment strategies. Proc. Am. Thorac. Soc..

[36] Magnussen H., Disse B., Rodriguez-Roisin R., Kirsten A., Watz H., Tetzlaff K., Towse L., Finnigan H., Dahl R., Decramer M. (2014). Withdrawal of inhaled glucocorticoids and exacerbations of COPD. N. Engl. J. Med..

[37] Vogelmeier C., Worth H., Buhl R., Criee C.P., Lossi N.S., Mailander C., Kardos P. (2017). “Real-life” inhaled corticosteroid withdrawal in COPD: A subgroup analysis of DACCORD. Int. J. Chronic Obstr. Pulm. Dis..

[38] Singh D., Agusti A., Anzueto A., Barnes P.J., Bourbeau J., Celli B.R., Criner G.J., Frith P., Halpin D.M.G., Han M. (2019). Global Strategy for the Diagnosis, Management, and Prevention of Chronic Obstructive Lung Disease: The GOLD science committee report 2019. Eur. Respir. J..

[39] Wedzicha J.A., Banerji D., Chapman K.R., Vestbo J., Roche N., Ayers R.T., Thach C., Fogel R., Patalano F., Vogelmeier C.F. (2016). Indacaterol–glycopyrronium versus salmeterol–fluticasone for COPD. N. Engl. J. Med..

[40] Pascoe S., Barnes N., Brusselle G., Compton C., Criner G.J., Dransfield M.T., Halpin D.M.G., Han M.K., Hartley B., Lange P. (2019). Blood eosinophils and treatment response with triple and dual combination therapy in chronic obstructive pulmonary disease: Analysis of the IMPACT trial. Lancet Respir. Med..

