# Supplementary material for: Stable Clinical Course of Chronic Obstructive Pulmonary Disease Patients in the Era of Double Bronchodilator Therapy: A Single Referral Center Experience
Source: J Clin Med. 2020 Aug 6;9(8):2547. doi: 10.3390/jcm9082547 (PMC7465249; doi:10.3390/jcm9082547)
Supplement: Supplementary file 1 [file jcm-09-02547-s001.pdf]

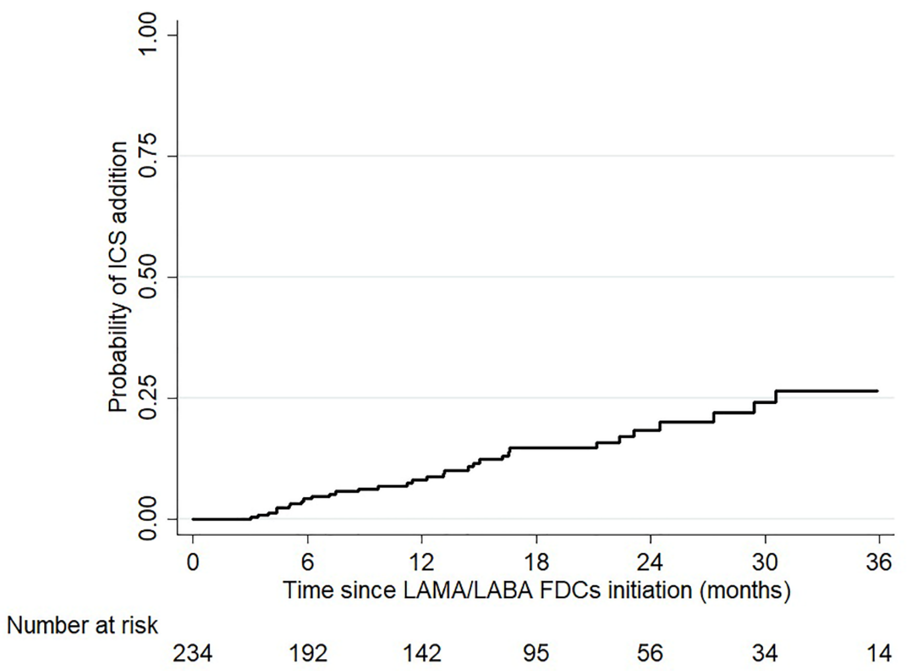

Figure S1. Kaplan-Meier curves for ICS addition in 234 patients who maintained LAMA/LABA fixed-dose combinations therapy for more than 90 days. FDC, fixed-dose combinations; ICS, inhaled corticosteroids; LABA, long-acting beta2-agonist; LAMA, long-acting muscarinic antagonist.

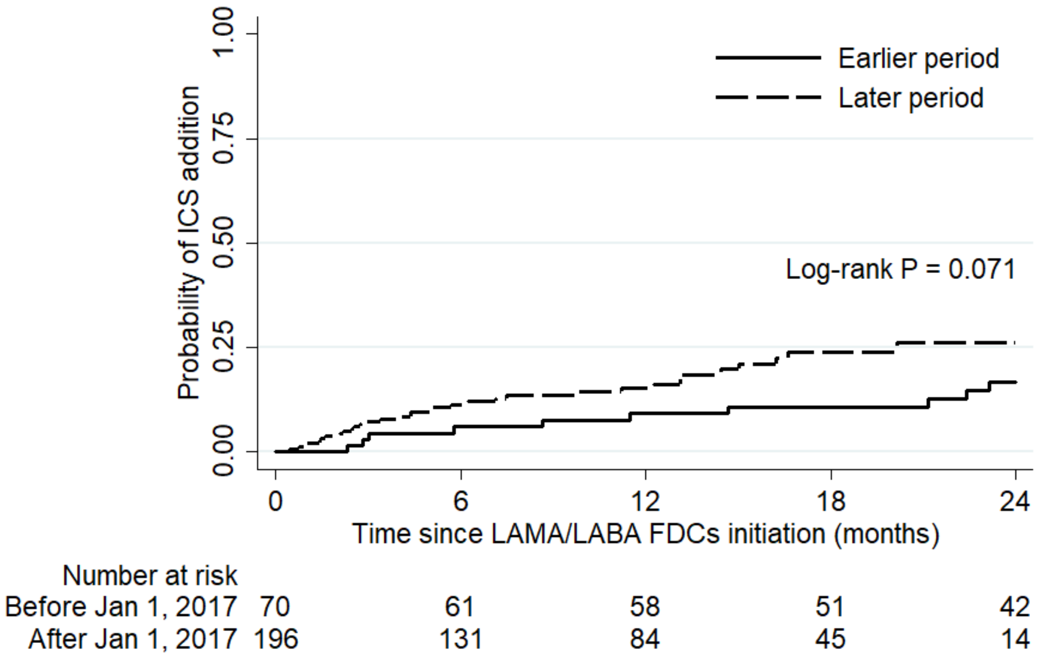

Figure S2. Kaplan-Meier curves for ICS addition in patients who started LAMA/LABA fixed-dose combinations therapy before and after January 1, 2017. FDC, fixed-dose combinations; ICS, inhaled corticosteroids; LABA, long-acting beta2-agonist; LAMA, long-acting muscarinic antagonist.

Table S1. Clinical factors associated with ICS addition after LAMA/LABA fixed-dose combinations therapy including only treatment naïve patients (N=108).

	Univariate analysis		Multivariate analysis	
	**Unadjusted HR (95% CI)**	**p-value**	**Adjusted HR (95% CI)**	**p-value**
Age, years	0.99 (0.93 – 1.06)	0.820	0.96 (0.90 – 1.03)	0.245
Male sex	2.59 (0.33 – 20.51)	0.369	4.32 (0.19 – 97.58)	0.358
Smoking, ever	1.38 (0.28 – 6.74)	0.689	0.34 (0.03 – 3.99)	0.391
BMI < 21(kg/m^2)	1.02 (0.29 – 3.61)	0.972	2.00 (0.44 – 9.19)	0.373
mMRC ≥ 2	1.72 (0.64 – 4.65)	0.285	2.23 (0.60 – 8.29)	0.230
CAT ≥ 10	1.37 (0.44 – 4.28)	0.587		
Exacerbation in the previous year*	7.40 (2.67 – 20.50)	**<0.001**	11.57 (3.24 – 41.38)	**<0.001**
GOLD grade ≥ 3†	1.10 (0.31 – 3.87)	0.885	1.10 (0.20 – 5.92)	0.912
DLco < 80% pred	0.71 (0.24 – 2.13)	0.544		
Positive BDR	2.39 (0.89 – 6.44)	0.085	3.14 (1.00 – 9.93)	0.051
Blood eosinophil count ≥ 300 (/μL)	0.83 (0.19 – 3.70)	0.804	2.05 (0.37 – 11.41)	0.412

* Exacerbation of moderate (hospital visit) or severe (emergency department or hospitalization) degree. † GOLD ≥ grade 3 (FEV_1 < 50% predicted). BDR, bronchodilator response; BMI, body mass index; CAT, COPD assessment test; CI, confidence interval; DLco, diffusing capacity for carbon monoxide; FEV_1, forced expiratory volume in 1s; GOLD, global initiative for chronic obstructive lung disease; HR, hazard ratios; ICS, inhaled corticosteroids; LABA, long-acting beta2-agonist; LAMA, long-acting muscarinic antagonist; mMRC, modified medical research council.

Table S2. Clinical factors associated with ICS addition after LAMA/LABA fixed-dose combinations therapy (N = 266).

	Univariate analysis		Multivariate analysis	
	**Unadjusted HR (95% CI)**	**p-value**	**Adjusted HR (95% CI)**	**p-value**
Age, years	1.01 (0.98 – 1.05)	0.460	0.99 (0.95 – 1.03)	0.594
Male sex	0.88 (0.32 – 2.46)	0.813	0.49 (0.09 – 2.64)	0.409
Smoking, ever	1.10 (0.39 – 3.06)	0.857	1.58 (0.90 – 8.29)	0.591
BMI < 21(kg/m^2)	0.97 (0.47–2.00)	0.927	0.70 (0.30 – 1.61)	0.399
mMRC ≥ 2	2.31 (1.28 – 4.16)	**0.005**	3.47 (1.77 – 6.78)	**<0.001**
CAT ≥ 10	1.69 (0.82 – 3.50)	0.157		
ICS withdrawal ahead of LAMA/LABA FDCs therapy	2.70 (1.47 – 4.94)	**0.001**	2.70 (1.32 – 5.53)	**0.007**
Exacerbation in the previous year*	3.39 (1.90 – 6.04)	**<0.001**	3.71 (1.98 – 6.95)	**<0.001**
GOLD grade ≥ 3†	1.80 (0.93–3.47)	0.080	1.37 (0.66 – 2.84)	0.403
DLco < 80% pred	1.87 (0.83–4.21)	0.132		
Positive BDR	1.36 (0.69 – 2.67)	0.376	1.76 (0.86 – 3.62)	0.122
Blood eosinophil count ≥ 300 (/μL)	0.91 (0.39 – 2.15)	0.833	1.12 (0.46 – 2.71)	0.800

* Exacerbation of moderate (hospital visit) or severe (emergency department or hospitalization) degree. † GOLD ≥ grade 3 (FEV_1 < 50% predicted). BDR, bronchodilator response; BMI, body mass index; CAT, COPD assessment test; CI, confidence interval; DLco, diffusing capacity for carbon monoxide; FDC, fixed-dose combination; FEV_1, forced expiratory volume in 1s; GOLD, global initiative for chronic obstructive lung disease; HR, hazard ratios; ICS, inhaled corticosteroids; LABA, long-acting beta2-agonist; LAMA, long-acting muscarinic antagonist; mMRC, modified medical research council.

Table S3. Changes in pulmonary function from baseline to the end of LAMA/LABA fixed-dose combinations therapy according to ICS addition afterwards (N = 261)*.

	ICS addition (-) (N = 211)*	ICS addition (+) (N = 47)	p-value
**Prebronchodilator FEV_1 (mL/year)**			
Annual change	44.96 (22.52, 67.40)	-21.96 (-88.46, 44.55)	
Average difference in annual change			
Crude	*Reference*	-66.92 (-137.10, 3.27)	0.062
Model 1	*Reference*	-66.62 (-136.90, 3.65)	0.063
Model 2	*Reference*	-66.41 (-138.31, 5.48)	0.070
**Prebronchodilator FEV_1 (% pred/year)**			
Annual change	2.57 (1.74, 3.41)	0.26 (-2.15, 2.67)	
Average difference in annual change			
Crude	*Reference*	-2.31 (-4.86, 0.23)	0.075
Model 1	*Reference*	-2.29 (-4.81, 0.24)	0.077
Model 2	*Reference*	-2.29 (-4.89, 0.30)	0.084

Values are mean (95% CI). * Five patients in non-ICS addition group had missing data. Model 1: Adjusted for age, sex, smoking history (never vs. ever), BMI (< 21 vs. ≥ 21 kg/m^2); Model 2: Further adjusted dyspnea (mMRC ≥ 2 vs. < 2), COPD severity (global initiative for chronic obstructive lung disease [GOLD] grade 1,2 vs. 3,4), previous ICS use, exacerbation in the previous year; BMI, body mass index; CI, confidence interval; GOLD, global initiative for chronic obstructive lung disease; ICS, inhaled corticosteroids; LABA, long-acting beta2-agonist; LAMA, long-acting muscarinic antagonist; mMRC, modified medical research council.
